# Lifespan map creation enhances stream restoration design

**DOI:** 10.1016/j.mex.2019.04.004

**Published:** 2019-04-06

**Authors:** S. Schwindt, G.B. Pasternack, P.M. Bratovich, G. Rabone, D. Simodynes

**Affiliations:** a Department of Land, Air and Water Resources, University of California at Davis, One Shields Avenue, Davis, CA 95616-8626, USA; b HDR, 2365 Iron Point Road, Suite 300, Folsom, CA 95630, USA; c Yuba Water Agency, 1220 F Street, Marysville, CA 95901, USA

**Keywords:** Lifespan map creation, Eco-morphology, Habitat enhancement, River management, Stream restoration, Sustainability, Feature lifetime

## Abstract

Research and engineering efforts are establishing a vast number of stream restoration planning approaches, design testing frameworks, construction techniques, and performance evaluation methods. A primary question arises as to the lifespan of stream restoration features. This study develops a framework to identify relevant parameters, design criteria and survival thresholds for ten multidisciplinary restoration techniques:

• Parameterize relevant features, notably, (1) bar and floodplain grading; (2) berm setback; (3) vegetation plantings; (4) riprap placement; (5) sediment replenishment; (6) side cavities; (7) side channel and anabranches; (8) streambed reshaping; (9) structure removal; and (10) placement of wood in the shape of engineered logjams and rootstocks.
• Identify survival thresholds for parameters, where the feature life ends when the threshold value is exceeded.
• Compare parameter thresholds with spatial data of topographic change and hydrodynamic forces as a result of hydrodynamic modelling of multiple discharges.

The discharge or topographic change rate that is related to the lowest (flood) return period spatially determines the feature’s lifespan in years.

## Graphical abstract

**Figure:**
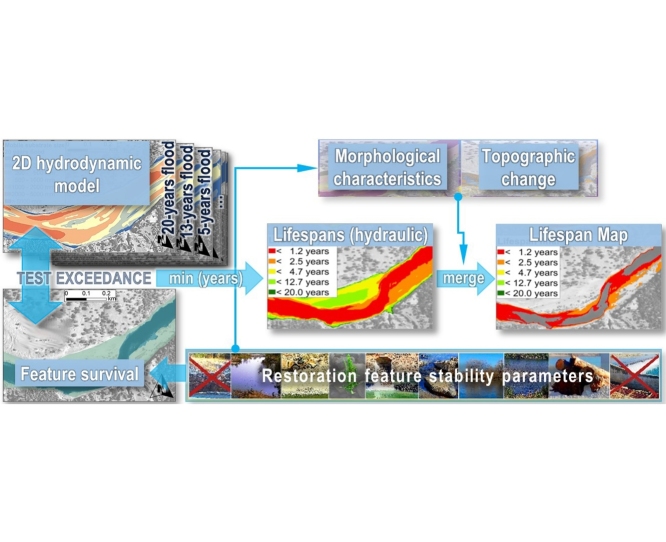

**Specifications Table**

**Table:** 

Subject Area:	*Engineering*
More specific subject area:	*River management, stream restoration and habitat enhancement*
Method name:	*Lifespan map creation*
Name and reference of original method:	*See main article* (*Schwindt et al.* [1])
Resource availability:	*See supplemental material in Schwindt et al.* [1]

## Method details

Schwindt et al. [1] review restoration features that apply to the river reach scale (10–100 times channel according to Pasternack and Wyrick [2]). Numeric hydro-morphodynamic stability criteria with threshold values for determining the feature longevity are identified for each of the ten considered features. Table 1 summarises the studied restoration features, applicable parameters and threshold values for every feature considered. In-channel morphological units are relevant parameter for some features. For instance, morphological units related to instable banks, such as “cutbank”, are relevant candidates for side cavities, which stabilize the banks and enhance the habitat. Wyrick and Pasternack [3] describe considerable in-channel morphological units and their assessment as a function of the flow depth and velocity.

**Table 1:** Summary of reach-scale restoration features, stability parameters and relevant threshold values (adapted from Schwindt et al. [1]).

Feature	Depth to water	Shear stress	Fill	Flow depth	Flow velocity	Froude number	Morph. unit	Scour
(name)	(m)	(–)	(m/year)	(m)	(m/s)	(–)	(string)	(m/year)
Bar & floodplain grading	2–4	0.047	na	na	na	na	yes	0.03
Berm setback	6–23	na	na	na	na	na	yes	na
Plants: Box Elder^a^	1–2	0.047	na	0.2·2	na	na	na	na
Plants: Cottonwood^a^	1.5–3	na	0.8·0.2·2	1.5·0.2·2	1	na	na	0.1·0.8·2
Plants: White Alder^a^	0.5–1.5	0.047	na	na	na	na	na	0.3
Plants: Willow^a^	1–1.5	0.1	na	0.2·2 + 0.1	na	na	na	0.1·0.8·2
Riprap	na	0.047	na	na	na	na	na	0.3
Sediment replenishment	na	0.047	na	na	na	na	na	na
Side cavities	na	na	0.3	na	na	na	yes	na
Side channels	Numerically not ascertainable							
Structure removal	Numerically not ascertainable							
Swale and backwater	na	0.047	0.03	na	0.03	na	yes	0.03
Wood	na	na	na	1.7·0.6	na	1	yes	na

a Hypotheses: Minimum stem height = 2 m, Planting depth = 80% of stem height.

Some features lack numerically quantifiable hydro-morphodynamic stability criteria, and therefore, lifespan maps cannot be developed for side channels or structural removal.

The particular threshold values compared with discharge-dependent values from the numerical 2D hydrodynamic models indicate the survival of features on maps. The modelled discharges correspond to flood return periods of, for example, 1, 5, 10 and 20 years, which serve for estimating the feature lifespan. The values for restoration feature stability thresholds are compared against 2D modelling derived rasters of at each discharge using GIS software (ESRI, 2018. ArcGIS Desktop: Release 10.6. or QGIS, 2019. QGIS 3.4.). Such comparisons spatially indicate where survival thresholds of a particular feature are exceeded.

In some cases, multiple parameters determine the feature lifespan, which requires the combination of several lifespan maps to determine the optimum location of a feature.

**Fig. 1:**
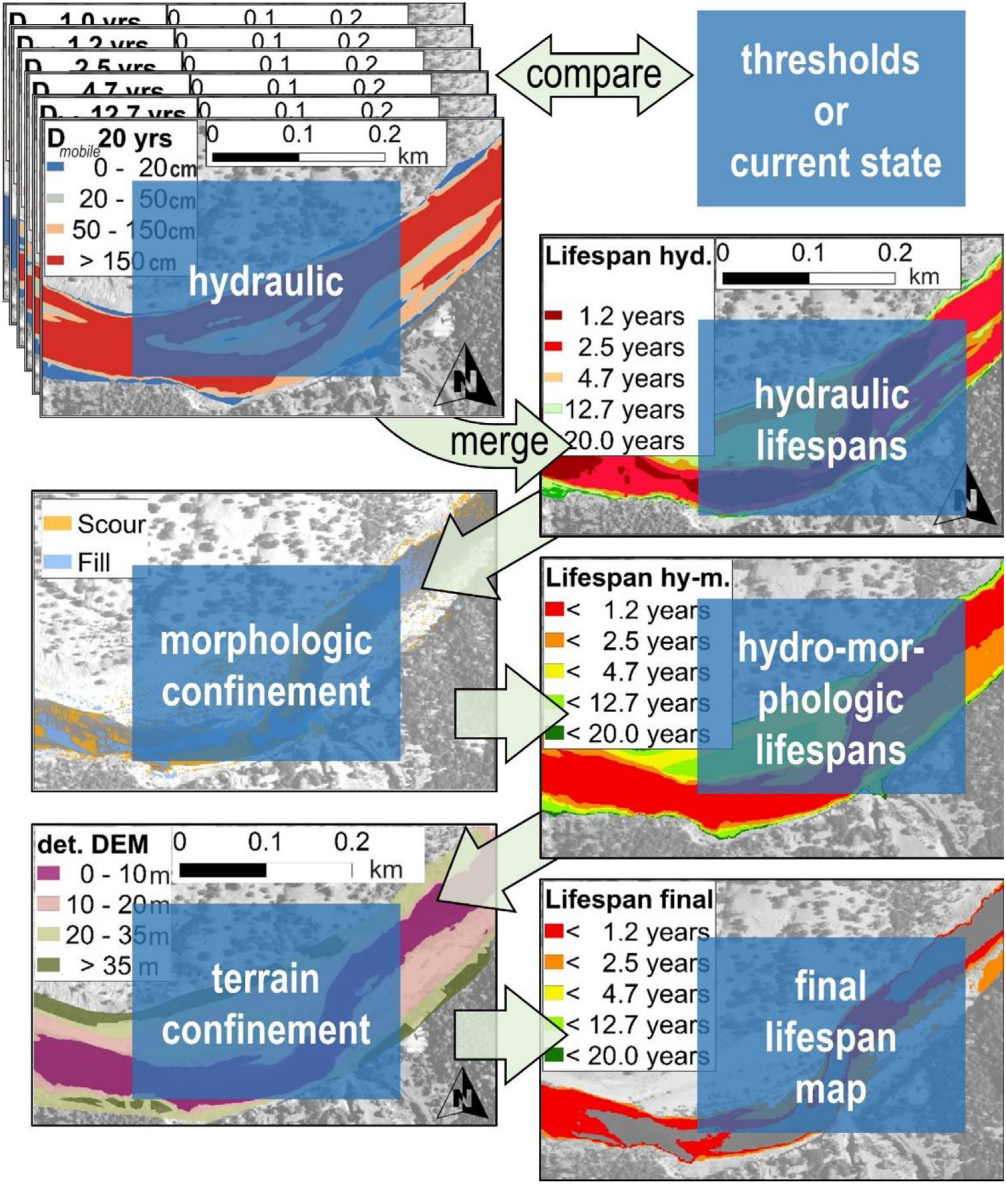
Exemplary procedure for the creation of lifespan maps. First, the present grain sizes are compared with the theoretically mobile grain sizes according to 2D modelling results. The smallest discharge with the lowest return period in years imposes the hydraulic feature lifespan. Second, topographic change rates are vetted against the hydraulic lifespan rates. If the annual erosion rate exceeds the erosion/deposition threshold of a particular feature at a pixel, this pixel’s lifespan is assigned a value of less than one year. Third, terrain confinements such as the depth to the groundwater table are applied to exclude non-sense regions. For example, plantings require the proximity to the groundwater, but many plant species do not support stagnant moisture neither (adapted from Schwindt et al. [1]).

Fig. 1 exemplarily illustrates the procedure for obtaining lifespan maps based on the discharge-dependent grain mobility (*D_mobile_*) compared with the observed grain size. Jackson et al. [4] provide a method for determining the surface grain size of large surfaces. The grain mobility maps result from applying Map Algebra tools (ESRI, 2018. ArcGIS Desktop: Release 10.6.) to the 2D model outputs of each of the considered flood discharges. The comparison of these maps with the present substrate grain sizes indicates the mobile surface related to the flood discharges. Merging these maps produces a hydraulic lifespan map, where the smallest discharge that mobilises grains is the limiting value. The amalgamation of multiple mobility frequency maps with rasters delineating the morphological applicability (scour/fill, morphological units) add the morphological component. Finally, the hydro-morphologic lifespan maps are matched with potential terrain confinements such as the depth to the groundwater table to produce what we denominate a “lifespan map” for every feature (adapted from Schwindt et al. [1]).

## Supplementary material and/or additional information

Please refer to the supplemental material of Schwindt et al. [1] for more details on feature planning, stability criteria and detailed calculation hints. Moreover, this supplemental material provides a comprehensive list of databases with ecologically relevant native plants for many regions in the world.
